# Insights into Reproductive Immunology and Placental Pathology, 2nd Edition

**DOI:** 10.3390/ijms27125529

**Published:** 2026-06-18

**Authors:** Dariusz Szukiewicz

**Affiliations:** Department of Biophysics, Physiology & Pathophysiology, Faculty of Health Sciences, Medical University of Warsaw, 02-004 Warsaw, Poland; dariusz.szukiewicz@wum.edu.pl

This Special Issue, the second one dedicated to reproductive immunology and placental pathology, is a response to the need to summarize the latest trends in research topics in this field [1]. The dynamic development of the immunopathology of pregnancy, especially a more detailed understanding of maternal–fetal interactions ensuring the proper course of implantation and subsequent placentation, has resulted in this update taking place after just two years [2].

In terms of gestational trophoblastic disease (GTD), hydatidiform moles or molar pregnancies are a rare complication of pregnancy, diagnosed in approximately 1 in 1000 to 1200 pregnancies in Western countries, and are much more common in the Far East [3,4]. Due to a genetic error during the fertilization of the oocyte, the abnormally growing daughter tissue contains an excess of paternal genetic material [5]. A distinction is made between a complete hydatidiform mole (CHM; the DNA is entirely paternal, and no embryo forms), when an empty oocyte is fertilized by one or two sperm, and a partial mole (PHM; an abnormal embryo begins to form), which occurs when a viable egg is fertilized by 2 sperm, leading to a triploid genome with maternal and paternal DNA [6]. In both types of molar pregnancies, the placental tissue grows rapidly, forming abnormal, fluid-filled cysts with the characteristic appearance of a grape-like cluster. Uterine enlargement that is rapid and inappropriate for gestational age is accompanied by extremely high levels of chorionic gonadotropin (hCG), severe nausea and vomiting (hyperemesis), and painless vaginal bleeding [7]. Histopathological diagnosis of a hydatidiform mole is crucial because molar pregnancies carry a significant risk of developing into persistent gestational trophoblastic neoplasia (GTN), including malignant choriocarcinoma [8]. A definitive tissue diagnosis directly determines patient monitoring, enabling early detection and successful treatment of malignancy [9,10]. However, relying solely on morphological assessment for histopathological differential diagnosis between CHM and PHM, as well as dysfunctional trophoblasts in miscarriage (e.g., hydropic abortus), is widely recognized as insufficient and prone to errors due to some common and overlapping morphological features [11]. Therefore, diagnostic reproducibility in molar pregnancies requires the introduction of auxiliary techniques [12,13]. For example, confirmation of the absence (negative immunostaining) of the p57 protein, which is encoded by the maternally imprinted CDKN1C gene, is useful for supporting the diagnosis of CHM, which does not contain maternal genetic material [14]. Molecular genotyping in the form of Short Tandem Repeat (STR) or microsatellite polymorphism analysis is also a valuable auxiliary technique because of the following:

- It may confirm that all alleles are strictly paternal and lack maternal alleles in CHM;
- By revealing the triploid genotype of cells with a unique 2:1 ratio of paternal/maternal genetic contribution, a diagnosis of PHM can be made;
- It can immediately rule out a molar pregnancy by identifying maternal alleles in the tissue in the case of non-molar abortion [15,16].

Flow cytometry for DNA ploidy analysis is a rapid technique that measures the total nuclear DNA content of individual cells within a population. As an auxiliary technique, it facilitates the distinction of diploid CHM cells from triploids, which is indicative of PHM [17]. An alternative technique for DNA ploidy analysis is in situ hybridization (ISH), both chromogenic (CISH) and fluorescent (FISH), which allows for the direct counting of chromosomes in placental tissue and the detection of the presence of triploidy in the case of PHM [18,19].

It is clear that providing an accurate diagnosis, guiding clinical follow-up, and determining the risk of GTN currently require pathologists to use histological evaluation in combination with immunohistochemistry and molecular genotyping [20,21].

Once the blastocyst is implanted, the trophoblast invades the spiral arteries, and low-resistance blood flow is created within the developing utero-placental unit; appropriate changes in the immune system of pregnant women, known as maternal–fetal immune tolerance, are necessary [22]. Analyses of possible causes of recurrent implantation failure (RIF) and early-onset preeclampsia (PE) have recently linked insufficient adaptation of the immune system to immune checkpoint networks within peripheral blood and the endometrium during its decidualization [23,24]. Immune checkpoints act as a molecular “weakener” of immune system activity, which prevents the rejection of the embryonic allograft by maternal immune cells, enables the invasive growth of extravillous cytotrophoblasts, and simultaneously preserves the pregnant woman’s ability to fight infections [25,26]. Key checkpoint pathways include the programmed cell death protein 1 (PD-1)/programmed cell death ligand 1 (PD-L1), cytotoxic T-lymphocyte associated protein 4 (CTLA-4) and T-cell immunoreceptor with Ig, and immunoreceptor tyrosine-based inhibitory motif (ITIM) domain (TIGIT)/CD226 (DNAX Accessory Molecule-1 [DNAM-1]) axes [25]. In women with RIF and PE, abnormal expression of immune checkpoint molecules in peripheral blood lymphocytes and in decidual immune cells, which disrupts maternal–fetal immune tolerance through the maintenance of high levels of the cytotoxic activities of natural killer (NK) cells, cytotoxic T cells and T helper 1 (Th1)/T helper 17 (Th17) cells toward the embryo, has been demonstrated [27,28,29]. Research into immune checkpoint pathways as therapeutic targets is ongoing, aiming at developing highly personalized immunotherapies for patients experiencing RIF or PE [30,31].

Galectins are a family of evolutionarily conserved soluble proteins, including 12 distinct genes/proteins in humans that specifically recognize and bind β-galactose-containing carbohydrates and lipids [32]. They form multivalent “galectin–glycan lattices” that cross-link glycoproteins on the cell surface with extracellular matrix (ECM) molecules. The action of galectins can be attributed to the function of “signalosomes”, which fine-tune cellular communication in health and disease [33,34]. This drives localized actin reorganization, cell spreading, and the assembly of focal adhesions, all of which influence cytoskeletal architecture [35]. Moreover, by participating in the spatial organization of tissue, galectins play a key role in placentation, not only ensuring a healthy maternal–fetal connection by promoting embryo implantation but also being vital regulators of immune responses (establishing maternal–fetal immune tolerance), trophoblast invasion and angiogenesis [36,37,38,39,40]. Most galectins are abundantly expressed within the maternal–fetal interface and placenta, and the expression of individual galectins is strictly controlled at the individual stages of the development of the utero-placental unit, which is reflected in quantitative and qualitative changes in the content of these proteins within the differentiating trophoblast (Figure 1).

The dysregulation of galectins is strongly correlated with serious obstetrical complications, such as miscarriages and preeclampsia [36,37]. Recent studies have indicated that abnormal galectin signaling plays an important role in the pathomechanism of placental dysfunction associated with gestational diabetes mellitus (GDM) [41]. Aberrant carbohydrate metabolism in GDM causes significant disruption of glucose homeostasis and lipid metabolism, resulting in various N-glycosylation features [46]. Changes in glycocodes, manifested as shifts in the placental N-glycome, changes in the ratio of glycoforms to N-glycosylated proteins, or the required concentrations of glycan-binding proteins to ensure trophoblast migration and invasion, are accompanied by abnormal placental morphology and changes in its function, including nutritional imbalances in placental transport [47]. The clinical importance of these disorders continues to increase because the global prevalence of GDM has increased significantly in recent years. Current data show that hyperglycemia occurs in approximately one in six pregnancies, with GDM accounting for approximately 85% of those cases. The increasing trend in the incidence of GDM is primarily driven by delayed childbearing, escalating rates of pre-pregnancy obesity, and changing diagnostic criteria [48,49]. However, further research is necessary to determine whether disturbances in placental galectin expression occur secondary to metabolic changes in GDM or whether these disturbances are a primary factor in the pathomechanism of GDM-complicated pregnancy. This will help determine whether galectins collected from blood, which are also present in placental tissue (e.g., Gal-1, Gal-3, and Gal-9), may be valuable prognostic biomarkers during pregnancy complicated by GDM [41].

Heat shock proteins (HSPs) are molecular chaperones composed of amino acid chains folded into specific structures that protect cells and assist protein folding [50]. The placenta expresses several key families of HSPs, categorized by their molecular weight. Since the placenta is particularly susceptible to hypoxia, oxidative stress and inflammation, the expression of HSPs in its tissue is strongly upregulated to maintain protein homeostasis (proteostasis) within the maternal–placental–fetal unit [51]. Placental HSPs are currently being investigated as highly promising diagnostic biomarkers and therapeutic targets for major pregnancy-related complications. Abnormal HSP expression—such as the dysregulation, upregulation, or depletion of specific HSP families (e.g., HSP27, HSP60, HSP70, HSP90, and HSPA5)—has been consistently demonstrated in several high-risk obstetric and placental ischemic diseases, including early pregnancy loss/miscarriage, PE, fetal growth restriction (FGR), spontaneous preterm birth, chorioamnionitis and placental abruption [52].

During the Epithelial–Mesenchymal Transition (EMT), stationary epithelial cells lose their tight attachments and polarity, transforming into highly motile, invasive mesenchymal cells [53]. This process of extensive biochemical and structural reprogramming is critical for embryonic development, wound healing, and cancer metastasis [54]. Essential steps occurring during embryogenesis, such as gastrulation, organ formation and neural crest generation, rely heavily on EMT. Moreover, the phenomenon of blastocyst attachment to the decidualized endometrium, a critical second phase of human implantation, requires precise modulation of EMT in the outer trophoblast layer (trophoectoderm), which acts as the direct physical and molecular bridge between the embryo and the uterus (trophoblast cells and uterine epithelium) [55]. As specialized epithelial cells, trophoblasts in the steady state exhibit their characteristic features, forming tight, polarized sheets of cells with a distinct top (apical) and bottom (basal), held together by strong intercellular junctions and anchored to a basement membrane [56]. Understanding the EMT is vital for developing early pregnancy therapies aimed at improving endometrial receptivity, maternal–fetal immune tolerance and placental vascularization [57,58].

Numerous recent studies on the immunopathology of pregnancy have consistently included regulatory T cells (Tregs) [59]. These specialized immune cells suppress effector T cells to prevent fetal rejection of the developing fetus, which acts as a semi-allograft containing paternal antigens. In addition to establishing maternal–fetal immune tolerance at the level of the fetal–placental unit, T-cell dysfunction is also analyzed in relation to immune-mediated pregnancy complications (such as PE and recurrent miscarriage) and altered susceptibility to infections [60]. Research is ongoing into the possibility of therapeutic interventions aimed at modulating Treg activity during pregnancy. Studies in animal models and the few clinical trials that have been initiated have shown promising results in the treatment of pregnancy-induced hypertension through the enhancement of Treg activity. However, considering the multifactorial pathomechanisms of the development of hypertension as a pregnancy complication and the complex and unclear regulatory mechanisms of Tregs, translating in vitro and animal model findings into effective clinical therapies is a constant challenge [61]. Finally, T cell-mediated immune dysregulation that leads to pro-inflammatory conditions during pregnancy may have a profound impact on newborn health. For example, acute and chronic inflammation during pregnancy may predispose the offspring to common neurodevelopmental disorders such as autism spectrum disorder (ASD), attention deficit hyperactivity disorder (ADHD) or Tourette syndrome (TS) [62].

The five original research papers and three review articles included in this Special Issue cover the topics mentioned above. Reading them will help you update your knowledge in the field of reproductive immunology, especially immunology at the maternal–fetal interface, gain insight into cutting-edge investigations, and inspire your own research.

## Figures and Tables

**Figure 1:**
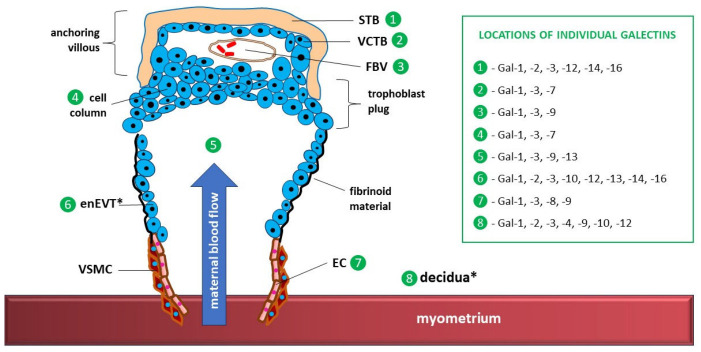
The expression pattern of galectins at the level of the maternal–fetal interface and placenta. Adopted from [41]. The strength of this expression and the localization of galectins are clearly developmentally regulated and dependent on trophoblast differentiation [37,38,39,40,42,43,44,45]. EC—endothelial cell; enEVT—endovascular extravillous trophoblast; STB—syncytiotrophoblast; VCTB—villous cytotrophoblast; VSMC—vascular smooth muscle cell; FBV—fetal blood vessel. * including immune cells.
